# NAM-BSA: A Bulked Segregant Analysis Method Using a NAM Population

**DOI:** 10.3390/plants15152335

**Published:** 2026-07-29

**Authors:** Shuangshuang Luo, Chi Liu, Yu Zeng, Xiuzhong Xia, Zongqiong Zhang, Baoxuan Nong, Can Chen, Rui Feng, Hui Guo, Danting Li, Xinghai Yang

**Affiliations:** Guangxi Key Laboratory of Rice Genetics and Breeding, Rice Research Institute, Guangxi Academy of Agricultural Sciences, Nanning 530007, China

**Keywords:** NAM-BSA technology, wild rice, cultivated rice, plant height, QTL

## Abstract

Common gene mapping approaches mainly include QTL-mapping, BSA-seq (QTL-seq) and GWAS. BSA-seq is designed to facilitate the mapping of quantitative trait loci (QTL) in a cost-effective and high-efficiency manner. In order to accommodate the diverse species-specific traits and population genetic architectures, researchers have developed a series of tailored BSA methodologies. In this study, using six wild rices (*Oryza rufipogon*) as donors and the elite cultivated rice Youzhan 8 (YZ8) as the recipient, we constructed a BC_4_F_8_ population through hybridization and backcrossing. These six wild rice introgression lines together constitute a nested association mapping (NAM) population. Upon genotyping 1819 lines of the NAM population for the *Sd1* gene, we found that among lines harboring the 383 bp deletion, 98.5–99.3% exhibited a low plant height (LP) phenotype, whereas 0.7–1.5% showed a high plant height (HP) phenotype. Based on this phenotypic segregation, we selected a total of 20 HP lines and 20 LP lines from six BC_4_F_8_ populations to form the H-bulk and L-bulk, respectively. Using BSA-seq, we identified a total of 33 significantly associated candidate intervals. One candidate interval located on chromosome 1 contains *D18*, a previously reported gene that regulates plant height. Furthermore, we preliminarily identified two major candidate genomic regions on chromosome 8 (4.60–5.76 Mb and 7.29–7.33 Mb). Integrating RNA-seq data, CAFRI-Rice online functional prediction and RT-qPCR validation, two key candidate genes, *LOC_Os08g09900* and *LOC_Os08g09000*, were selected for subsequent functional characterization. The results of this study indicate that the NAM-BSA technology has great potential to detect QTL associated with complex traits, which can provide a novel technical strategy for the genetic dissection of complex traits in rice.

## 1. Introduction

Rice is the staple food for more than half of the world’s population and plays a critical role in ensuring global food security [1]. However, long-term artificial selection has narrowed the genetic diversity of cultivated rice, resulting in reduced resistance to diseases, pests, and environmental stresses [2]. In contrast, wild rice has developed high genetic diversity and strong adaptability to diverse environments through long-term natural selection [3].

At present, a large number of elite genes related to stress resistance [4,5], disease resistance [6,7,8], and yield have been excavated from wild rice [9,10]. Nevertheless, wild rice possesses many unfavorable agronomic traits, which increases the difficulty of gene mining and restricts the efficient utilization of its germplasm resources, requiring further in-depth exploration. Common methods for genetic dissection mainly include linkage analysis-based approaches and linkage disequilibrium-based approaches. Traditional biparental linkage analysis can be used to map rare allelic variants. However, biparental populations have a limited number of recombination events, which only enable the identification of large chromosomal intervals at the megabase scale, resulting in low mapping resolution [11]. Genome-wide association study (GWAS) is a method based on linkage disequilibrium, which can be applied to both natural populations and artificial populations. GWAS based on natural populations leverages historical recombination events accumulated over long-term population evolution, and combined with high-throughput molecular marker genotyping technology, it greatly improves mapping efficiency [12]. Nevertheless, natural populations are generally characterized by population stratification and cryptic relatedness among individuals, which readily trigger false positive results in association tests [13]. A nested association mapping (NAM) population is a type of multiparent population, constructed by crossing and backcrossing one recurrent parent with multiple donor parents [13]. Multiparent populations have advantages over biparental populations as they produce additional recombination breakpoints and increase the allelic diversity and power of QTL detection [14]. Following the successful application of NAM in maize, the NAM strategy has been rapidly adopted for genetic studies in other crops, including soybean [15], rice [16], and wheat [17]. Despite the prominent advantages of NAM populations, it remains an urgent technical challenge in the field of plant genetics and breeding to develop efficient gene mapping methods compatible with their complex multiparent genetic structure.

Bulked segregant analysis (BSA) was first reported in 1991 and has greatly accelerated the detection of major quantitative trait loci (QTL) in lettuce [18]. BSA has undergone rapid development in plant science, evolving from traditional genetic mapping approaches to multi-omics-integrated strategies. Early technologies such as SHOREmap [19], X-QTL [20], and NGM [21] significantly improved the efficiency of QTL mapping. In the era of high-throughput sequencing, innovative methods such as QTL-seq [22] and MutMap [23] have enabled higher-throughput and more precise gene mapping through genome-wide SNP analysis. Recently, emerging approaches including PCAMP [24], GradePool-seq [25], and DeepBSA [26] have further expanded the scope and depth of BSA-based studies. However, methods such as SHOREmap and X-QTL require relatively large sample sizes; QTL-seq and DeepBSA require the construction of bi-parental segregating populations; and MutMap is primarily applicable to the identification of monogenic traits in mutant lines. BSA and its derivative techniques are mostly applied in biparental populations, and there are very few studies on gene mapping using BSA in multiparent populations [27].

Plant height is an important agronomic trait affecting rice yield formation [28]. The rice *Sd1* gene encodes GA20 oxidase, and its mutation results in a semi-dwarf phenotype, serving as a key gene for rice yield improvement [29]. Loss-of-function mutation in *D18*, a critical gene involved in gibberellin biosynthesis, causes severe dwarfism in rice [30]. Rice plant height is a polygenic quantitative trait influenced by both genetics and environmental factors [28]. Despite the identification of multiple genes regulating rice plant height, the underlying regulatory mechanisms remain incompletely elucidated, and numerous unknown loci and genes await further identification and characterization.

In this study, a NAM population consisting of 1819 lines was constructed using the cultivated rice variety *YZ8* as the recurrent parent and six wild rice accessions as donor parents. This population incorporates allelic variations from multiple parental lines, greatly enriching the genetic diversity of the population. Phenotypic evaluation revealed that plant height in the NAM population varied by more than 90 cm (84–195 cm), which provides excellent experimental material for constructing extreme phenotypic bulks in BSA. To efficiently mine unknown genes regulating plant height within the NAM population, we developed the NAM-BSA analytical method. This method integrates the high genetic resolution of multiparent populations and the mapping advantages of BSA technology, and is expected to facilitate the identification of more candidate genes regulating rice plant height. The results of this study are expected to provide a reference for elucidating the potential regulatory mechanisms of rice plant height, and offer feasible strategies for the efficient development and utilization of elite wild rice germplasm resources.

## 2. Results

### 2.1. Construction of the NAM Population and Analysis of Agronomic Traits

Wild rice is characterized by high genetic diversity and strong environmental adaptability and therefore represents a valuable genetic reservoir for rice improvement. The wild rice materials used in this study were collected from different regions at home and abroad, and the parents exhibited abundant genetic diversity. The basal leaf sheaths of P1 and P3 are purple, while those of P2, P5, P6 and P7 (YZ8) are white. The grains of the six wild rice accessions all possess long purple awns; their sterile lemmas are lanceolate, both lemma and palea are covered with short pubescence and turn black at maturity, with a red seed coat. In contrast, P7 bears awnless grains with lanceolate sterile lemmas. Its lemma and palea are covered with short pubescence, the lemma and palea are brownish yellow at maturity, and the seed coat is white (Figure S1). The grain length of each parent ranged from 7.6 to 8.9 mm, grain width varied from 2.1 to 2.8 mm, and the 1000-grain weight was between 14.02 and 20.52 g.

**Figure 1:**
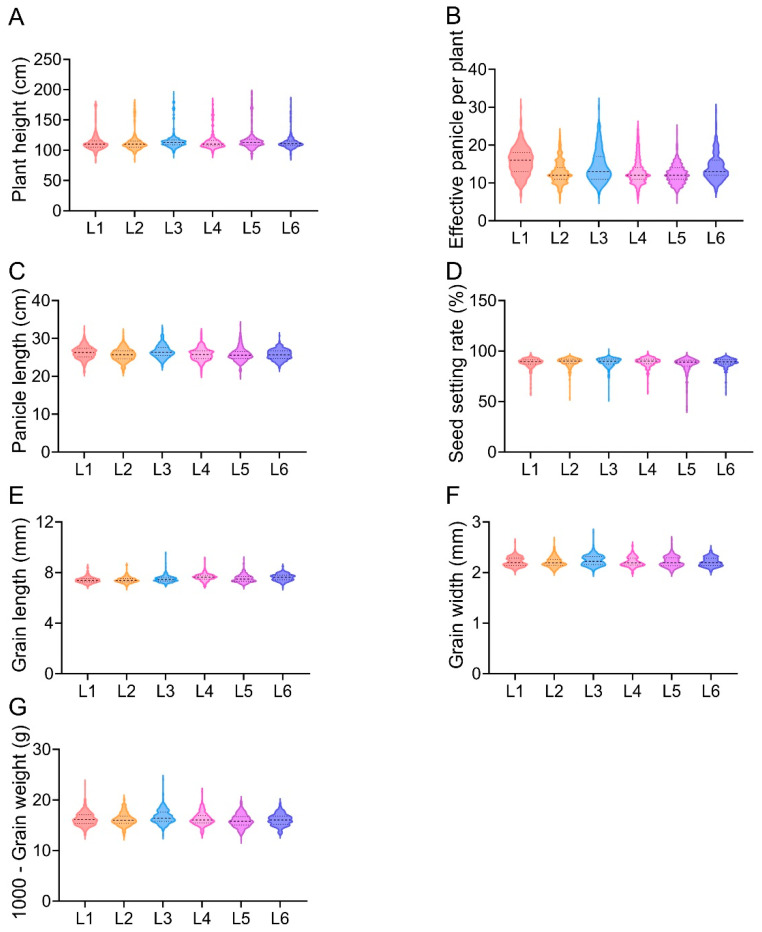
Agronomic trait analysis of the NAM population. (**A**) Plant height; (**B**) Effective panicle; (**C**) Panicle length; (**D**) Seed setting; (**E**) Grain length; (**F**) Grain width; (**G**) 1000-Grain weight. L1, L2, L3, L4, L5, and L6 represent the BC_4_F_8_ populations derived from Chaling wild rice, Dongxiang wild rice, Yuanjiang wild rice, Cambodian wild rice, Guangxi wild rice 1, and Guangxi wild rice 2 as donor parents, respectively.

To efficiently identify genes control complex traits in wild rice, the cultivated rice variety *YZ8* was selected as the common recurrent parent. Six wild rice accessions—P1, P2, P3, P4, P5, and P6—were used as donor parents to generate F_1_ hybrids in the early season of 2012. From 2013 to 2014, the progeny from each combination were continuously backcrossed to the recurrent parent P7 for four generations, with individuals retaining prominent wild rice traits being preferentially selected at each generation for backcrossing. In the late season of 2014, 160 BC_4_F_1_ combinations obtained from each subpopulation were planted in the field, with one row (10 plants per row) for each combination. At maturity, two plants with distinct phenotypic differences were selected from each row for individual seed harvesting, yielding a total of 320 lines. From 2015 to 2017, these 320 lines from each BC_4_F_1_ population were subjected to single-seed descent for six consecutive generations to achieve homozygosity. Finally, in 2018, a NAM population consisting of six subpopulations (L1, L2, L3, L4, L5, and L6) was established (Figures S1 and S2). The sizes of the six BC_4_F_8_ subpopulations were 279, 300, 314, 302, 306, and 300 lines, respectively, totaling 1819 lines (Table S1). To further characterize the phenotypic features of the NAM population, seven agronomic traits including plant height, effective panicle number and panicle length were phenotypically evaluated. The coefficients of variation in plant height and effective panicle number were the highest (Figure 1A,B; Table S2), while the remaining five traits exhibited relatively narrow phenotypic ranges (Figure 1C–G; Table S2). A cluster analysis of the entire NAM population using phenotypic data for seven traits revealed that the population can be divided into five subpopulations, each of which includes lines derived from different wild rice lineages.

### 2.2. Genotypic Analysis of the Sd1 Gene in the NAM Population

Based on the agronomic trait analysis of the NAM population, plant height exhibited a particularly wide range of variation. Previous studies have shown that the rice *Sd1* gene regulates gibberellin biosynthesis by encoding GA20 oxidase. A deletion of a 278 bp fragment spanning exons 1 and 2, together with a 105 bp intronic fragment, results in loss of GA20 oxidase function and leads to a dwarf phenotype [31]. To investigate the genetic basis underlying the large variation in plant height observed in the NAM population, we designed a primer pair, XianSd1, targeting a 383 bp deletion in the *Sd1* gene, based on established knowledge of *Sd1*-mediated control of rice plant height. In theory, an amplification product of 606 bp corresponds to a tall-plant phenotype (HP; >125 cm), whereas a 223 bp product corresponds to a dwarf-plant phenotype (LP; ≤125 cm). However, our analysis revealed that individuals producing the 223 bp *Sd1* amplification product displayed both HP and LP phenotypes. Although the majority of these plants (98.5–99.3%) exhibited the expected dwarf phenotype, a small proportion (0.7–1.5%) showed a tall phenotype. These results indicate that the observed variation in plant height within the NAM population cannot be fully explained by functional defects in the *Sd1* gene alone, suggesting the involvement of additional genetic factors.

### 2.3. Development of the NAM-BSA Method

**Figure 2:**
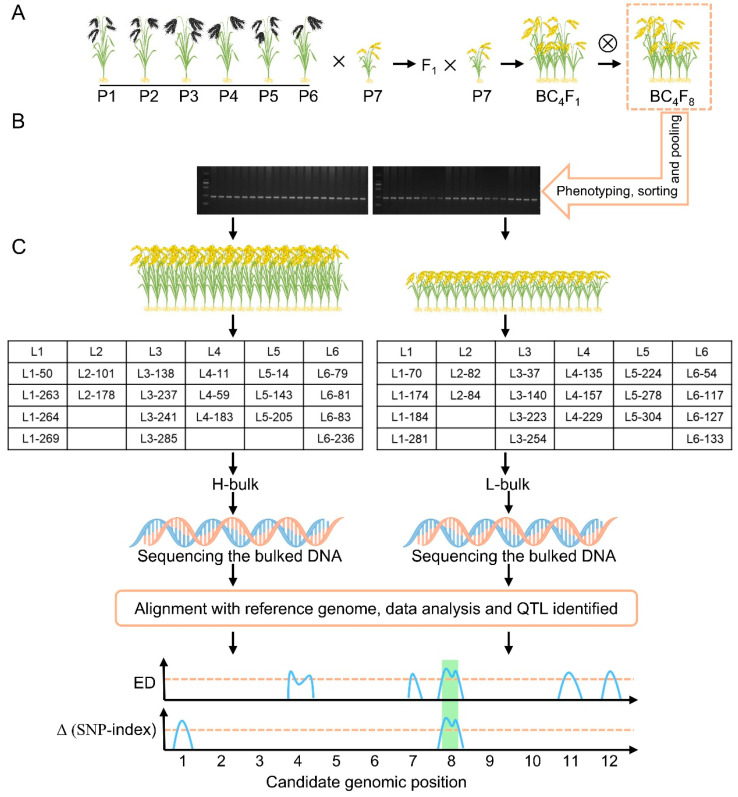
Workflow of the NAM-BSA method for QTL mapping of rice plant height. (**A**) P1, P2, P3, P4, P5, P6, and P7 represent Chaling wild rice, Dongxiang wild rice, Yuanjiang wild rice, Cambodian wild rice, Guangxi wild rice 1, Guangxi wild rice 2, and *YZ8*, respectively. (**B**) The PCR bands shown correspond to a 223 bp fragment. (**C**) L1–L6 denote the six BC_4_F_8_ subpopulations. The green regions indicate candidate genomic intervals that were consistently identified by two independent statistical methods in the BSA. The orange dashed lines indicate the threshold values for ED and Δ(SNP-index), which were set at 0.08 and 0.17, respectively. The regions outlined by the blue curves correspond to the significant candidate genomic intervals as-sociated with the target trait on this chromosome.

To further investigate the genetic factors responsible for plant height variation among lines sharing the same *Sd1* genotype, we developed a novel QTL mapping strategy termed NAM-BSA (Figure 2). First, the NAM population was constructed by crossing six donor parents (P1–P6) with the recurrent parent P7, followed by four generations of backcrossing and six generations of single-seed descent (Figure 2A). Next, PCR amplification using the XianSd1 primer set was performed to screen the NAM population. From this population, 20 HP individuals and 20 LP individuals that all carried the same *Sd1* genotype (the 223 bp deletion) were selected (Figure 2B). Genomic DNA was extracted from each individual and pooled in equal amounts to construct the high plant height bulk (H-bulk) and low plant height bulk (L-bulk) samples for BSA. We performed association analysis using two methods, Euclidean distance (ED) and Δ (SNP-index). Genomic regions with significant associations detected by both methods were ultimately selected as the optimal candidate intervals for the target trait (Figure 2C).

### 2.4. Application of NAM-BSA for Identifying Genomic Regions Associated with Rice Plant Height

According to the manufacturer’s instructions, genomic DNA was extracted from samples used for BSA and mixed in equal amounts to generate two extreme bulks, namely the H-bulk and the L-bulk. Illumina high-throughput sequencing generated 164,284,766 and 178,151,994 clean reads from the H-bulk and L-bulk, respectively. In both datasets, more than 95% of bases had Q30 scores, ensuring the reliability and accuracy of major QTL mapping. Clean reads were aligned to the reference genome, resulting in mapping rates of 98.25% for the H-bulk and 98.24% for the L-bulk. SNP and InDel variants were subsequently identified using the HaplotypeCaller method implemented in the GATK software. A total of 2,274,739 SNPs were detected in the H-bulk and 2,229,156 SNPs in the L-bulk. After filtering low-quality variants, 1,126,885 high-quality SNPs were retained. In addition, 482,165 InDels were identified in the H-bulk and 474,742 InDels in the L-bulk, and after quality filtering, 242,456 high-quality InDels were obtained.

**Figure 3:**
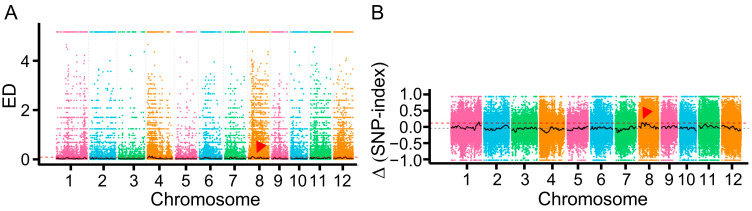
BSA of rice plant height. (**A**) Genome-wide ED distribution of plant height; (**B**) Δ(SNP-index) plot for rice plant height trait. The red arrows in (**A**,**B**) indicate candidate genomic regions that were co-localized. The black wavy line represents the fitted curve for H-bulk and L-bulk, while the red dashed line indicates the threshold line. The thresholds for (**A**,**B**) are 0.08 and 0.17, respectively.

ED analysis was used on SNPs from the H-bulk and L-bulk. The association threshold for this analysis was set as the median plus three standard deviations (median+3SD) of the fitted values at all loci, which yielded a threshold value of 0.08 [32]. A total of seven significantly associated candidate regions were identified (Figure 3A). Δ (SNP-index) analysis was conducted, with the 99% confidence interval of the fitted curve set as the significance threshold of 0.17 [33]. Five significantly associated candidate regions were detected in total (Figure 3B). We used ED analysis on the identified InDels. Analysis with the median+3SD yielded a threshold of 0.18, and we identified 18 significant candidate regions (Figure S3A). Δ (InDel-index) analysis was performed with the 99% confidence interval of the fitted curve set as the significance threshold of 0.19. Six significant candidate regions were identified (Figure S3B). Both ED and Δ (SNP-index) analyses identified candidate intervals on chromosome 8 (4.60–5.76 Mb, 7.29–7.33 Mb; Figure 3A,B), confirming two stable QTL on chromosome 8. These QTL provide a reference for subsequent studies on the regulatory mechanisms of rice plant height.

### 2.5. Gene Analysis Within Candidate Genomic Regions

Functional annotation of genes located within these candidate genomic regions led to the identification of one previously reported plant height–related gene, *d18*, on chromosome 1 (Figure S3A; Table S3) [30]. We further analyzed the genes located within the two candidate intervals that showed significant associations with both the ED and Δ (SNP-index) methods. A total of 180 candidate genes were identified in the interval Chr8: 4.60–5.76 Mb, including 41 transposon- and retrotransposon-related genes, 62 genes with unknown functions, and 77 expressed genes. The interval Chr8: 7.29–7.33 Mb contained only four genes, among which three were retrotransposon-related genes and one was an expressed gene (Table S4).

### 2.6. Further Analysis of Candidate Genes Using RNA-Seq

**Figure 4:**
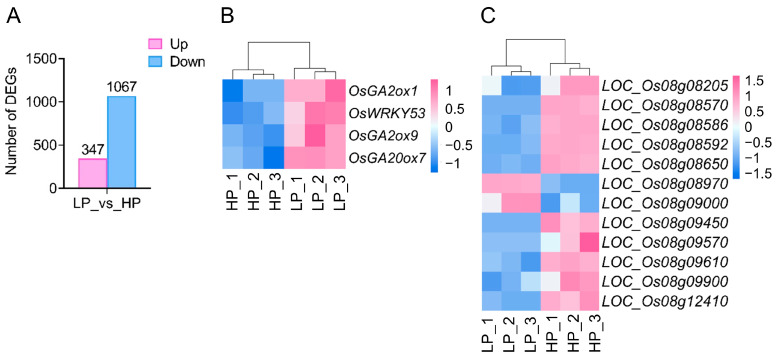

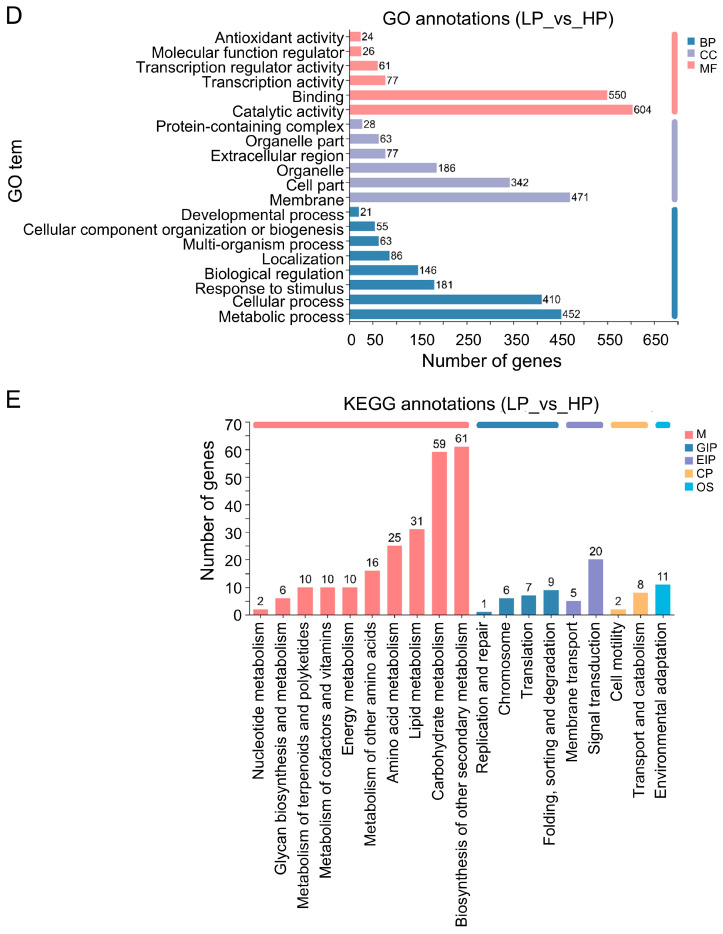
Transcriptomic analysis of extreme plant height pools to uncover genes regulating rice plant height. (**A**) Number of upregulated and downregulated DEGs identified; (**B**) Genes known to be associated with plant height were detected; (**C**) Expression heatmap of candidate genes located within the QTL interval on rice chromosome 8; (**D**) GO term classification of DEGs (LP_vs_HP); (**E**) KEGG pathway classification of DEGs (LP_vs_HP).

We performed RNA-seq analysis on HP and LP lines, yielding a total of 48.55 Gb of clean data. Each sample generated more than 6.26 Gb clean data, with Q30 ratios exceeding 94.43% (Table 1). Comparative transcriptome analysis between high-plant-height and low-plant-height groups (HR_vs_LR) identified 1414 differentially expressed genes (DEGs), including 1067 upregulated and 347 downregulated genes (Figure 4A). Among these DEGs, four previously reported plant height–related genes were identified: *OsGA2ox1* [34], *OsGA2ox7* [35], *OsGA2ox9* [36], *OsWRKY53* [37]. Compared with LP plants, all four genes showed significantly lower expression levels in HP plants (Figure 4B).

Among the identified DEGs, 12 genes were found to reside in the candidate genomic regions Chr8: 4.60–5.76 Mb and Chr8: 7.29–7.33 Mb (Figure 4C). Of these, ten genes showed significantly higher expression levels in HP than in LP, including *LOC_Os08g08586*, *LOC_Os08g08592*, *LOC_Os08g09610*, *LOC_Os08g08570*, *LOC_Os08g09450*, *LOC_Os08g08650*, *LOC_Os08g12410*, *LOC_Os08g08205*, *LOC_Os08g09570*, *and LOC_Os08g09900.* Two germin-like protein genes, *LOC_Os08g08970* and *LOC_Os08g09000*, were expressed at significantly lower levels in HP lines than in LP lines. We found that the expression patterns of the two germin-like protein genes were consistent with those of four previously identified plant height-related genes in HP and LP individuals (Figure 4B,C). The candidate genes were predicted via the online tool CAFRI-Rice (https://cafri-rice.khu.ac.kr/inspector; accessed on 15 February 2026), and functional redundancy was identified between *LOC_Os08g09900* and *LOC_Os05g27730* (*OsWRKY53*). Tian et al. [37] reported that *OsWRKY53* positively regulates brassinosteroid signaling in rice, and that overexpression of this gene leads to reduced plant height. The functional redundancy between *LOC_Os08g09900* and *OsWRKY53* suggests that *LOC_Os08g09900* may perform a similar biological role. Based on these results, the germin-like protein genes *LOC_Os08g08970* and *LOC_Os08g09000*, and the WRKY family gene *LOC_Os08g09900* were identified as the most promising candidate genes.

Gene Ontology (GO) and Kyoto Encyclopedia of Genes and Genomes (KEGG) enrichment analyses were performed for the DEGs. GO analysis showed that DEGs were mainly enriched in terms related to catalytic activity, binding, and cellular processes (Figure 4D). KEGG pathway analysis indicated that DEGs were significantly enriched in pathways such as metabolism of terpenoids and polyketides, metabolism of cofactors and vitamins, and signal transduction, all of which may be associated with the regulation of plant height (Figure 4E).

### 2.7. RT-qPCR Analysis of Candidate Gene Expression

**Figure 5:**
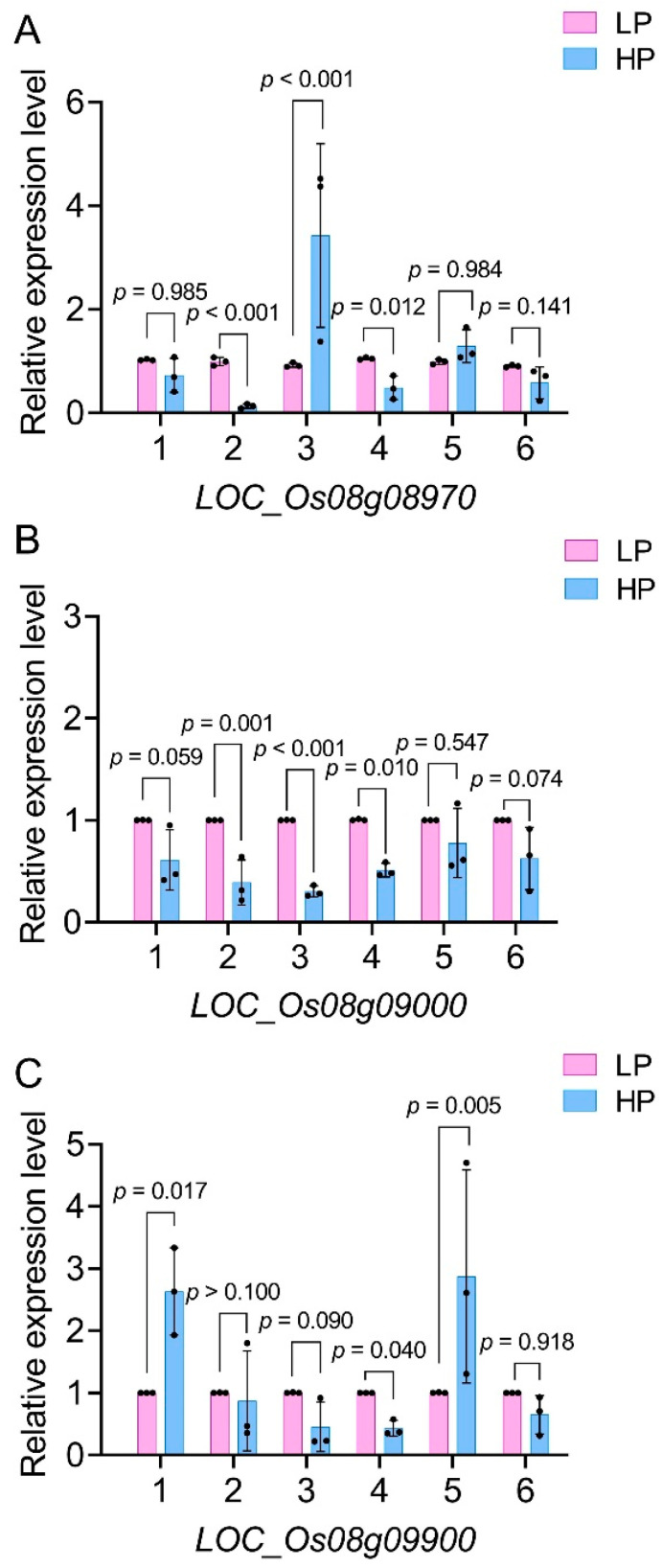
RT-qPCR analysis of candidate gene expression. (**A**) Transcriptional level analysis of *LOC_Os08g08970* in HP and LP lines; (**B**) Transcriptional level analysis of *LOC_Os08g09000* in HP and LP lines; (**C**) Transcriptional level analysis of *LOC_Os08g09900* in HP and LP lines. LP indicates low-plant-height lines, and HP indicates high-plant-height lines. 1, 2, 3, 4, 5 and 6 represent the comparisons between the six groups of LP lines and HP lines, respectively. Error bars represent the standard deviation (SD) of the mean values (*n* = 3).

The transcriptional levels of the three selected candidate genes were examined in six HP lines and six LP lines using RT-qPCR. The results showed that the germin-like protein gene *LOC_Os08g09000* exhibited a consistent expression pattern across all six HP/LP line pairs. Specifically, the transcript level of this gene was downregulated in all HP lines compared with the corresponding LP lines (Figure 5B), with significant downregulation observed in five of the six comparisons. This pattern is consistent with the RNA-seq results, which also showed downregulation of this gene in HP lines. In contrast, the other two candidate genes did not show a clear or consistent transcriptional trend across the multiple HP and LP line pairs (Figure 5A,C).

## 3. Discussion

This study proposes a novel QTL mapping strategy designated the NAM-BSA method, which integrates the strengths of NAM populations and BSA coupled with high-throughput sequencing. Compared with conventional QTL mapping approaches, the NAM-BSA method exhibits several distinct advantages.

First, a multi-parent NAM population was constructed in this study using the cultivated rice cultivar *YZ8* as the recurrent parent and multiple wild rice accessions as donor parents. Compared with bi-parental populations, NAM populations feature abundant genetic diversity, abundant recombination events, and wide applicability [14,38,39]. Second, the introduction of wild rice donor parents endows this population with abundant favorable wild rice allelic variations. Wild rice generally exhibits undesirable agronomic traits including loose plant architecture, tall plant height prone to lodging, low seed setting rate, and easy seed shattering, which greatly hinders phenotypic evaluation using wild rice materials alone. In contrast, NAM lines have been genetically improved in plant architecture, plant height, seed setting rate and other key agronomic traits relative to wild rice accessions, substantially reducing the difficulty of phenotypic identification. This population serves as an excellent genetic resource for mining superior genes governing complex traits from wild rice. Third, compared with GWAS, QTL mapping via the NAM-BSA method does not require large-scale phenotypic characterization of the entire population, which shortens the experimental cycle and reduces research costs [27]. In addition, conventional BSA, QTL-seq, DeepBSA and other related approaches all rely on specifically developed segregating populations [22,26,27]. By contrast, the NAM-BSA strategy eliminates the need for constructing additional segregating populations. Only extreme lines with target trait phenotypes need to be selected from the pre-established NAM population to generate the high- and low-phenotype bulks required for BSA sequencing. This strategy avoids the time required for re-establishing a segregating population and preserves the low-cost advantage of classical BSA. Nevertheless, this method still has several limitations: mapping resolution is restricted due to genetic linkage, and conventional BSA analysis exhibits weak anti-interference capacity when applied to highly heterozygous genomes.

In this study, the NAM-BSA strategy was utilized to perform QTL mapping on plants carrying the dwarfing genotype of *Sd1* yet exhibiting HP and LP phenotypes. A total of 33 significant candidate genomic regions were identified. Among these regions, the previously reported plant height-related gene *D18* was detected on chromosome 1 [30]. Furthermore, two candidate intervals (4.60–5.76 Mb and 7.29–7.33 Mb) on chromosome 8 were simultaneously identified via two association analysis methods, namely ED and Δ (SNP-index). The genes in these two candidate intervals deserve further analysis. To further mine candidate genes, we performed RNA-seq analysis on two groups of lines with extreme plant height phenotypes. Among the obtained DEGs, four known plant height-related genes were identified: *OsGA2ox1* [34], *OsGA2ox7* [35], *OsGA2ox9* [36], and *OsWRKY53* [37]. The expression levels of these genes were all significantly downregulated in HP plants compared with LP plants. A total of 12 DEGs were identified within the two core candidate intervals on chromosome 8 in this study, including *LOC_Os08g08586*, *LOC_Os08g08592*, *LOC_Os08g09610*, *LOC_Os08g08570*, *LOC_Os08g09450*, *LOC_Os08g08650*, *LOC_Os08g12410*, *LOC_Os08g08205*, *LOC_Os08g09570*, *and LOC_Os08g09900.* Among them, *LOC_Os08g08205*, *LOC_Os08g08570*, *LOC_Os08g08586*, *LOC_Os08g08592*, *LOC_Os08g08650*, *LOC_Os08g09570*, and *LOC_Os08g09610* are expressed-protein genes; *LOC_Os08g08970* and *LOC_Os08g09000* encode germin-like proteins; *LOC_Os08g09450* encodes an E3 ubiquitin ligase; *LOC_Os08g09900* belongs to the WRKY transcription factor family; and *LOC_Os08g12410* encodes a pectinesterase. Functional analysis of these 12 genes was conducted via the CAFRI-Rice database, and one WRKY family gene, *LOC_Os08g09900*, was found to share functional redundancy with the known plant height gene *OsWRKY53*. Previous studies have demonstrated that overexpression of *OsWRKY53* reduces plant height in rice [37]. Further RT-qPCR verification revealed that the expression level of *LOC_Os08g09900* was significantly lower in multiple HP lines relative to LP lines. Accordingly, we hypothesize that this gene may exert functions similar to *OsWRKY53* and acts as a vital candidate gene governing plant height variation in this population. In addition, two germin-like protein genes, *LOC_Os08g08970* and *LOC_Os08g09000*, were screened out in this study, whose expression patterns in HP and LP lines were highly consistent with those of the four known plant height-related genes. RT-qPCR analysis of these two genes showed that the transcript level of *LOC_Os08g09000* was significantly lower in HP lines than in LP lines. Most of the cloned germin-like protein genes have been mainly investigated for disease resistance functions to date [40,41], and only *OsGLP1* has been verified to regulate rice plant height; downregulated expression of this gene leads to a semi-dwarf phenotype in T2 rice progenies [42]. Whether *LOC_Os08g09000* shares similar functions with *OsGLP1* warrants further investigation. In addition to *LOC_Os08g09900* and *LOC_Os08g09000*, we note that *LOC_Os08g12410* (encoding a pectinesterase) also deserves consideration, as previous studies have demonstrated that pectinesterase genes are involved in cell wall loosening and the regulation of plant height [43,44].

## 4. Materials and Methods

### 4.1. Parent Materials and NAM Population Construction

We selected cultivated rice variety *YZ8* as the female parent. Chaling wild rice (P1), Dongxiang wild rice (P2), Yuanjiang wild rice (P3), Cambodian wild rice (P4), Guangxi wild rice 1 (P5), and Guangxi wild rice 2 (P6) served as the shared male parents. Between 2012 and 2018, NAM population consisting of 6 subpopulations (L1, L2, L3, L4, L5, and L6) was established through hybridization, backcrossing, and selfing (Figures S1 and S2 and Table S1). The six BC_4_F_8_ populations comprised 279, 300, 314, 302, 306, and 300 lines respectively, totaling 1819 lines.

### 4.2. Analysis of Agronomic Traits and Sd1 Genotypes in NAM Population

Phenotypic measurements of seven agronomic traits, including plant height, effective panicle number, panicle length, seed setting rate, grain length, grain width and 1000-grain weight, were performed on 1819 lines of the NAM population using a Wanshen seed analyzer (Hangzhou Wanshen Testing Technology Co., Ltd., Hangzhou, China), electronic balance and straight ruler. Statistical analysis showed that plant height possessed a relatively high coefficient of variation and exhibited wide phenotypic ranges in all six subpopulations; hence, this trait was selected for further in-depth analysis.

Previous studies have demonstrated that the rice *Sd1* gene encodes GA20 oxidase and regulates gibberellin biosynthesis. A 278 bp deletion in exons 1 and 2, together with a 105 bp intronic fragment deletion, impairs the function of GA20 oxidase and consequently results in semi-dwarf plant architecture. Based on the above 383 bp fragment deletion, a functional specific primer pair XianSd1 targeting the *Sd1* gene was designed in the present study. The primer XianSd1 was used to genotype the *Sd1* in all 1819 lines.

### 4.3. Dissecting Plant Height-Related Genomic Regions Through NAM-BSA Method

Theoretically, lines with a 223 bp amplification fragment generated by primer pair XianSd1 should exhibit a semi-dwarf phenotype. Nevertheless, lines carrying this 223 bp fragment within the NAM population segregated into both low and high plant height phenotypes. Moreover, the number of these atypical tall lines differed among the six BC_4_F_8_ subpopulations, and most subpopulations harbored only 2 to 4 such individuals. Based on the phenotypic variations observed among *Sd1* loss-of-function individuals, 4, 2, 4, 3, 3 and 4 HP and the same number of LP individuals were selected from subpopulations L1, L2, L3, L4, L5 and L6, respectively. At the jointing stage, uniformly growing plants were chosen, and the elongated internodes of main culms were sampled. All tissue samples were snap-frozen in liquid nitrogen and stored at −80 °C for subsequent experiments. Genomic DNA was extracted from each individual and mixed in equal amounts to construct the H-bulk and L-bulk, respectively (Figure 2C). Meanwhile, genomic DNA of the parent cultivar YZ8 was extracted as a control. All samples were delivered to Biomax Biotechnology Co., Ltd., Beijing, China for whole-genome resequencing and BSA.

DNA samples were sequenced on the Illumina platform to generate 150 bp paired-end reads. Raw sequencing data were processed using Fastp (version 0.21.0) to remove reads containing adapters, filter reads with an N content greater than 10%, and discard reads where more than 50% of bases possessed a Phred quality score below 10. The base error rate was calculated via the formula *Q_Phred_* = −10log_10_ ^(e)^. The clean reads were aligned to the Nipponbare reference genome (IRGSP-1.0) using bwa-mem2 (version 2.2) [45]. The resulting alignments were sorted and deduplicated with SAMtools (version 1.9) [46], followed by further filtering using GATK (version 3.8) [47] to obtain high-quality SNP and InDel variants. Subsequently, association analyses were performed using two independent algorithms: ED [32] and index-based methods [33]. Genomic regions that were significantly associated with the target trait by both algorithms were ultimately selected as high-confidence candidate intervals.

### 4.4. RNA-Seq and DEGs Analysis

Six HP lines (L1-50, L2-101, L3-138, L4-11, L5-14, L6-79) and six LP lines (L1-70, L2-82, L3-37, L4-135, L5-224, L6-54) were selected as experimental materials and cultivated under consistent growth conditions. All lines were sampled at the rice jointing stage; for each line, five uniformly growing individual plants were selected, and jointing tissues were collected separately from each plant. Total RNA was extracted from samples following the manufacturer’s instructions. The concentration and purity of RNA were detected using a Nanodrop 2000 spectrophotometer (Thermo Fisher Scientific, Wilmington, DE, USA), RNA integrity was verified by agarose gel electrophoresis, and the RQN value was determined using an Agilent 5300 instrument (Agilent Technologies Inc., Santa Clara, CA, USA). Qualified RNA samples were screened, and mRNA was enriched via the Oligo dT method for reverse transcription to synthesize cDNA. The in-group cDNA samples were mixed equally, and the mixture was divided into three equal aliquots for three independent sequencing runs. The libraries were subjected to paired-end sequencing on the Illumina platform to generate raw reads. After removing adapter sequences and low-quality reads from the raw data, high-quality clean reads were obtained and aligned to the Nipponbare reference genome (osa1_r7.asm). Transcript expression levels were quantified using the transcripts per million (TPM) method, and differential expression analysis was conducted using DESeq2 (version 1.10.1). Genes with |log_2_ (fold change) | ≥ 1 and *p* < 0.05 were defined as differentially expressed genes (DEGs). Hierarchical clustering analysis of DEGs was performed to explore expression patterns. In addition, the expression profiles of genes within the candidate intervals were used as supporting evidence for identifying genes associated with plant height. Gene Ontology (GO) enrichment analysis and Kyoto Encyclopedia of Genes and Genomes (KEGG) pathway analysis were carried out using Goatools (version 0.6.5) and Python SciPy (version 1.11.4) software, respectively.

### 4.5. RT-qPCR Analysis

Six HP lines and six LP lines were selected respectively; sampling was carried out at the jointing stage. Three biological replicates were used for each strain. Total RNA was extracted using the EASYspin Plant RNA Extraction Kit (Biomed, Wuhan, China), and reverse transcription was conducted with the First-Strand cDNA Synthesis and One-Step gDNA Removal Kit. RT-qPCR analysis was carried out on the CFX384 Touch Real-Time PCR System (Bio-Rad, Hercules, CA, USA). The PCR cycling conditions were as follows: initial denaturation at 95 °C for 2 min, followed by 40 cycles of 95 °C for 15 s, 58 °C for 30 s, and 72 °C for 30 s. The rice *Ubiquitin* (UBQ) was used as the internal reference gene for normalization [48], and the relative transcript levels of target genes were calculated via the 2^−ΔΔCt^ method [49]. The primers used for RT-qPCR are listed in Table S5.

### 4.6. Candidate Gene Analysis

In the BSA, candidate genomic regions that showed significant associations with the target trait across multiple computational methods were selected. Candidate genes within these intervals were retrieved from the rice genome database (https://rice.uga.edu/jb2/; accessed on 10 January 2026) and functionally annotated using information from the National Rice Data Center (https://www.ricedata.cn/gene/; accessed on 28 January 2026). Functional redundancy analysis of the candidate genes was subsequently performed using CAFRI-Rice (https://cafri-rice.khu.ac.kr/inspector; accessed on 15 February 2026). Finally, the expression levels of the candidate genes were validated by RT-qPCR analysis.

## 5. Conclusions

In this study, six wild rice germplasm accessions from home and abroad were crossed and backcrossed with the cultivated rice cultivar *YZ8* (recurrent parent) to construct a rice NAM population. BSA combined with two methods, ED and Δ (SNP-index), was performed on HP and LP lines for association mapping. A total of 33 significant candidate intervals were identified, among which the candidate interval on chromosome 1 harbors *D18*, a previously reported gene regulating plant height. Furthermore, two candidate intervals (4.60–5.76 Mb and 7.29–7.33 Mb) on chromosome 8 were simultaneously detected by both methods. Integrating RNA-seq data, online functional prediction and RT-qPCR validation, two key candidate genes, *LOC_Os08g09900* and *LOC_Os08g09000*, were screened out for subsequent functional characterization. The NAM-BSA strategy established in this research integrates the merits of multi-parent populations and BSA. It enables trait association mapping without developing conventional segregating populations separately, and at a relatively low cost, which may serve as a helpful method for the genetic dissection of complex agronomic traits in rice.

## Figures and Tables

**Table 1:** Sequencing data statistics.

Sample	Raw Data (Gb)	Clean Data (Gb)	Q20 (%)	Q30 (%)	GC Content (%)	Mapped Ratio (%)
HP_1	11.93	11.83	98.88	94.43	54.5	93.79
HP_2	7.35	7.30	99.37	96.81	51.94	95.08
HP_3	6.29	6.26	99.31	96.48	52.98	94.92
LP_1	10.08	10.01	98.89	94.47	54.54	93.89
LP_2	6.84	6.79	99.28	96.37	53.43	94.85
LP_3	6.40	6.36	99.27	96.35	53.3	94.64

## Data Availability

Data will be made available on request.

## Supplementary Materials

The following supporting information can be downloaded at: https://www.mdpi.com/article/10.3390/plants15152335/s1, Figure S1. Analysis of agronomic traits in seven parents. P1, P2, P3, P4, P5, P6, and P7 represent Chaling wild rice, Dongxiang wild rice, Yuanjiang wild rice, Cambodian wild rice, Guangxi wild rice 1, Guangxi wild rice 2, and *Youzhan* 8, respectively. The scale for the plant is 20 cm; the scale for the panicle is 5 cm; the scale for the leaf is 1 cm; the scale for the seed is 1 cm. Figure S2. NAM Population construction process. Figure S3. BSA of rice plant height. The red arrows in (B) indicate candidate genomic regions that were co-localized. The black wavy line represents the fitted curve for H-bulk and L-bulk, while the red dashed line indicates the threshold line. The thresholds for (A,B) are 0.18, and 0.19, respectively. Figure S4. Phylogenetic heatmap of *LOC_Os08g09900* with Anatomy expression database. The genes marked with red boxes are *LOC_Os08g09900* and *LOC_Os05g27730* (*OsWRKY53*), respectively. Table S1. Basic information of NAM parents. Table S2. Analysis of agronomic traits in the NAM population. Table S3. BSA-associated candidate genomic regions for plant height. Table S4. Candidate genes and gene annotation. Table S5. The primers which were used in this study.
