# Supplementary material for: NAM-BSA: A Bulked Segregant Analysis Method Using a NAM Population
Source: Plants (Basel). 2026 Jul 29;15(15):2335. doi: 10.3390/plants15152335 (PMC13468384; doi:10.3390/plants15152335)
Supplement: Supplementary file 1 [file plants-15-02335-s001.zip › plants-4439015-supplementary.pdf]

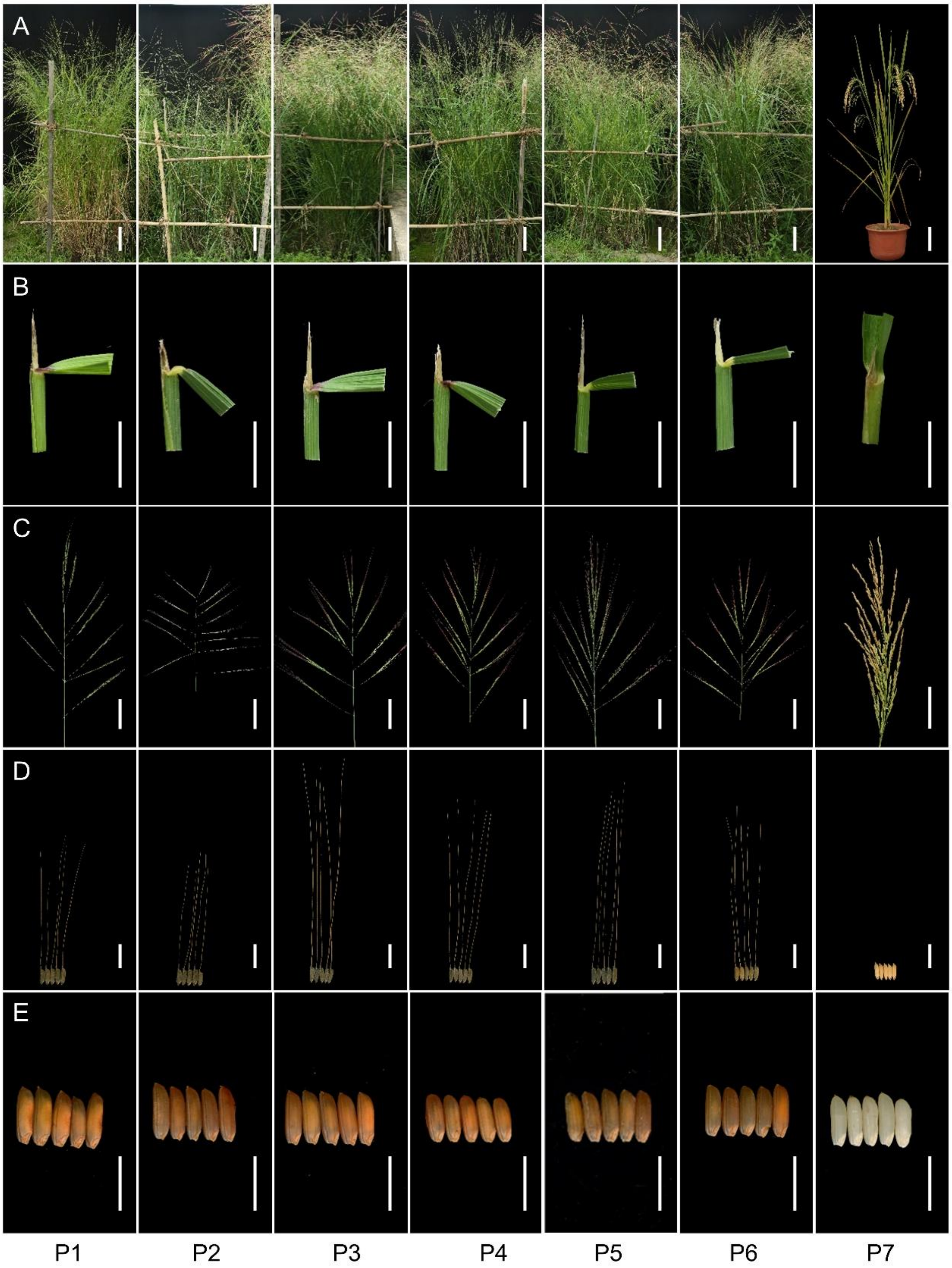


Supplementary Figure S1. Analysis of agronomic traits in seven parents. P1, P2, P3, P4, P5, P6, and P7 represent Chaling wild rice, Dongxiang wild rice, Yuanjiang wild rice, Cambodian wild rice, Guangxi wild rice 1, Guangxi wild rice 2, and *Youzhan* 8, respectively. The scale for the plant is 20 cm; the scale for the panicle is 5 cm; the scale for the leaf is 1 cm; the scale for the seed is 1 cm.

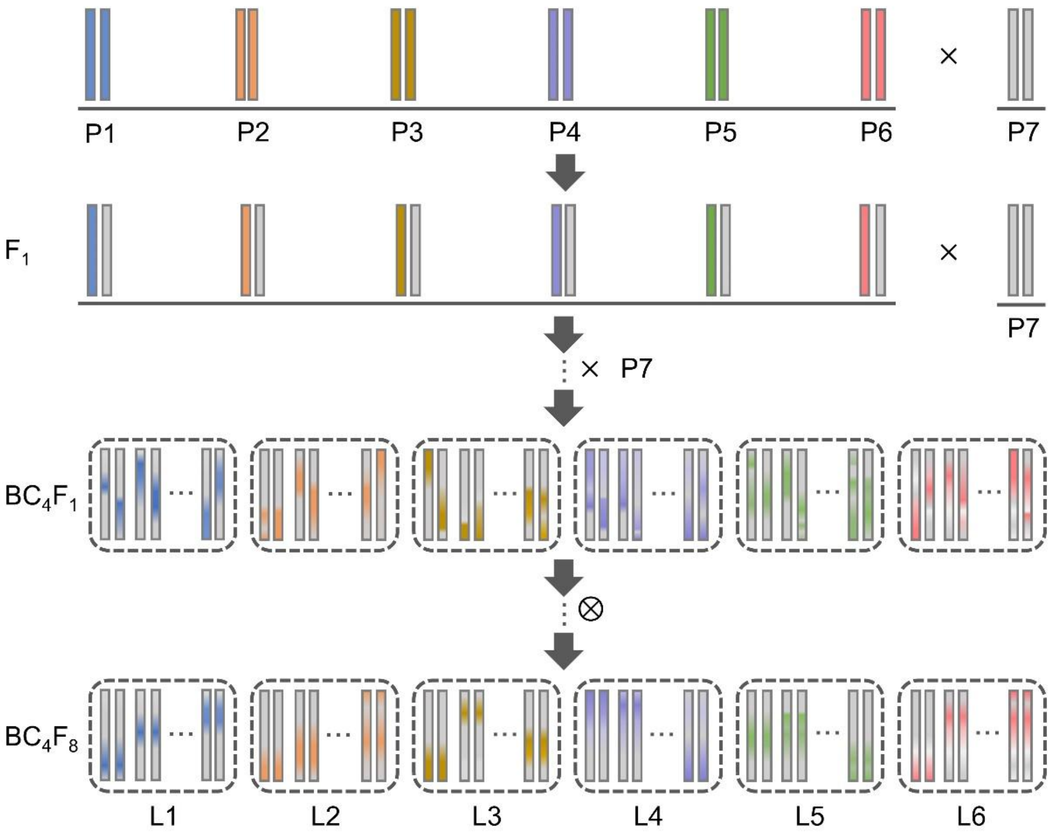


Supplementary Figure S2. NAM Population construction process. Detailed information of L1-L6 is shown in Table 1.

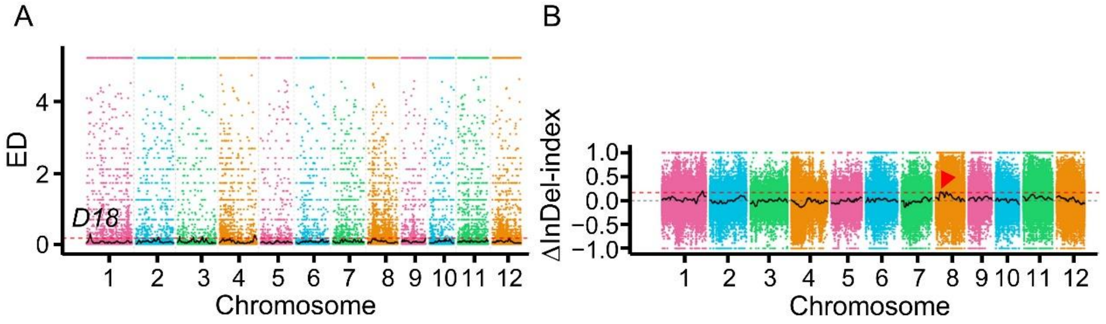


Supplementary Figure S3. BSA analysis of rice plant height. (A) Genome-wide ED distribution of plant height; (B) Δ(Indel-index) plot for rice plant height trait. The red arrows in (**B**) indicate candidate genomic regions that were co-localized. The black wavy line represents the fitted curve for H-bulk and L-bulk, while the red dashed line indicates the threshold line. The thresholds for (**A**, **B**) are 0.18, and 0.19, respectively.

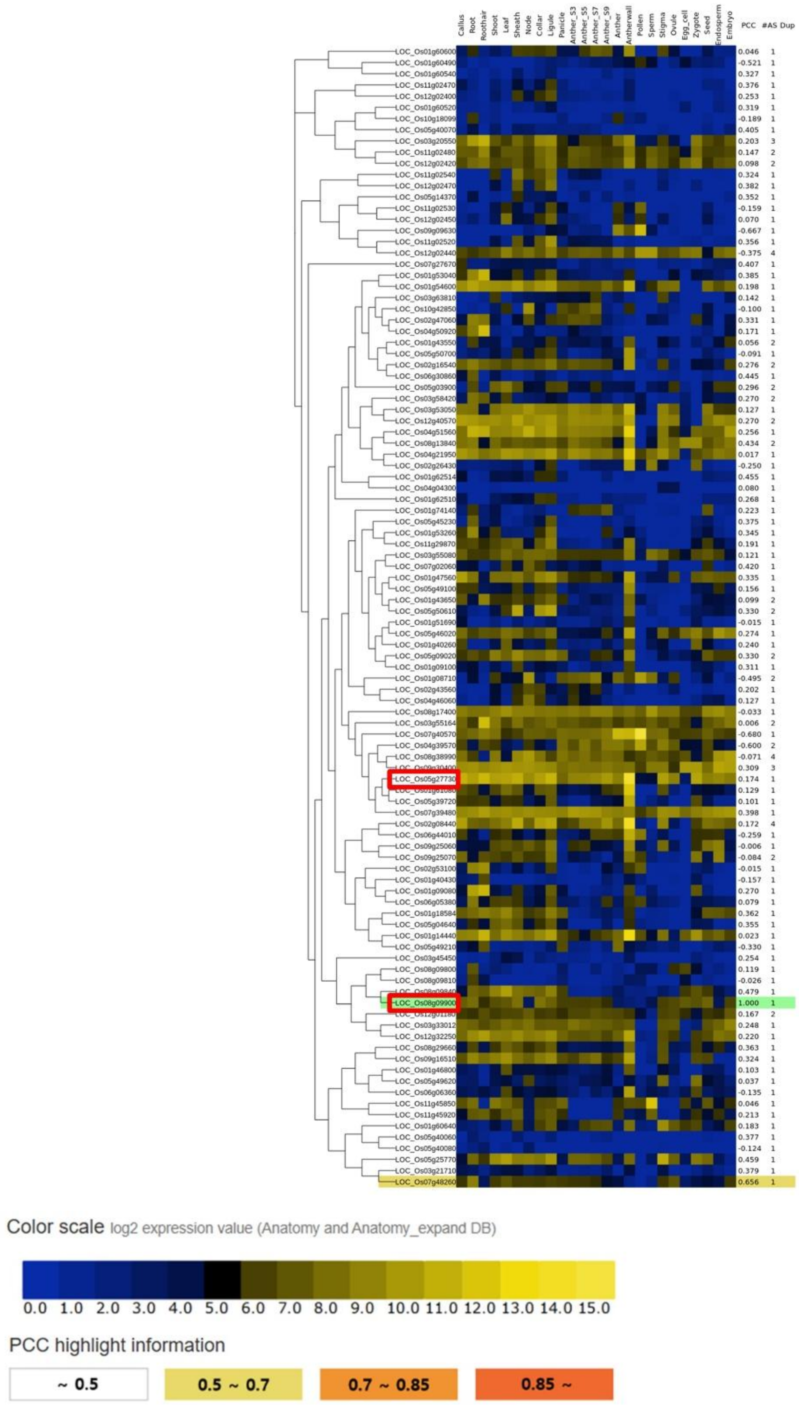


Supplementary Figure S4. Phylogenetic heatmap of *LOC_Os08g09900* with Anatomy expression database. The genes marked with red boxes are *LOC_Os08g09900* and *LOC_Os05g27730* (*OsWRKY53*), respectively.

**Supplementary Table S1 Basic information of NAM parents**

| Population number | Parental name | Parent type | Geographic origination | LSCB | AP | AC | GL (mm) | GW (mm) | SSH | LP | LC | SCC | 1000-GW (g) | Population size |
|---|---|---|---|---|---|---|---|---|---|---|---|---|---|---|
| L1 | Chaling wild rice | Male parent | Hunan | Purple | Yes | Purple | 8.9 | 2.8 | Lanceolate | Yes | Black | Red | 16.00 | 279 |
| L2 | Dongxiang wild rice | Male parent | Jiangxi | White | Yes | Purple | 8.5 | 2.4 | Lanceolate | Yes | Black | Red | 20.52 | 300 |
| L3 | Yuanjiang wild rice | Male parent | Yunnan | Purple | Yes | Purple | 8.5 | 2.5 | Lanceolate | Yes | Black | Red | 19.43 | 314 |
| L4 | Cambodian wild rice | Male parent | Cambodian | Purple | Yes | Purple | 7.6 | 2.4 | Lanceolate | Yes | Black | Red | 14.02 | 302 |
| L5 | Guangxi wild rice 1 | Male parent | Guangxi | White | Yes | Purple | 8.5 | 2.5 | Lanceolate | Yes | Black | Red | 18.5 | 306 |
| L6 | Guangxi wild rice 2 | Male parent | Guangxi | White | Yes | Purple | 7.9 | 2.4 | Lanceolate | Yes | Black | Red | 15.24 | 300 |
| P7 | Youzhan 8 | Female parent | Guangxi | White | No | No | 7.7 | 2.1 | Lanceolate | Yes | Yellow | White | 16.59 | |

LSCB: Leaf sheath color of the base; AP: Awn presence; AC: Awn color; GL: Grain length; GW: Grain width; SSH: Shape of the spikelet hull; LP: Lemma pubescence; LC: Lemma color;

**Supplementary Table S2 Analysis of agronomic traits in the NAM population**

| Trait | Min. | Max. | Mean | Standard deviation | Coefficient of variation (%) |
|---|---|---|---|---|---|
| Plant height (cm) | 84 | 195 | 114.4 | 15.18 | 13 |
| Effective panicle per plant | 6 | 30 | 13.8 | 3.56 | 26 |
| Panicle length (cm) | 20.1 | 33.6 | 26.0 | 1.67 | 6 |
| Seed setting rate (%) | 41.7 | 100 | 88.5 | 5.11 | 5 |
| Grain length (mm) | 6.8 | 9.5 | 7.5 | 0.28 | 3 |
| Grain width (mm) | 2.0 | 2.8 | 2.2 | 0.1 | 4 |
| 1000-Grain weight (g) | 12.8 | 24.1 | 16.3 | 1.29 | 7 |

**Supplementary Table S3 BSA-associated candidate genomic regions for plant height**

| Method | Chromosome | Start | End | Range size (Mb) | Plant height gene | Gene annotation |
|---|---|---|---|---|---|---|
| SNP-ED | 11 | 25,840,000 | 26,000,000 | 0.16 | | |
| SNP-ED | 12 | 15,600,000 | 17,280,000 | 1.68 | | |
| SNP-ED | 4 | 1,280,000 | 4,140,000 | 2.86 | | |
| SNP-ED | 4 | 5,330,000 | 7,320,000 | 1.99 | | |
| SNP-ED | 7 | 8,690,000 | 9,140,000 | 0.45 | | |
| SNP-ED | 8 | 16,490,000 | 17,180,000 | 0.69 | | |
| SNP-ED | 8 | 4,280,000 | 7,450,000 | 3.17 | | |
| Δ(SNP-index) | 1 | 38,750,000 | 40,520,000 | 1.77 | | |
| Δ(SNP-index) | 8 | 10,380,000 | 11,090,000 | 0.71 | | |
| Δ(SNP-index) | 8 | 11,130,000 | 11,140,000 | 0.01 | | |
| Δ(SNP-index) | 8 | 4,600,000 | 5,760,000 | 1.16 | | |
| Δ(SNP-index) | 8 | 7,290,000 | 7,330,000 | 0.04 | | |
| InDel-ED | 1 | 2,840,000 | 2,940,000 | 0.1 | | |
| InDel-ED | 1 | 2,970,000 | 4,450,000 | 1.48 | *D18* | Dwarf gene |
| InDel-ED | 1 | 4,480,000 | 4,600,000 | 0.12 | | |
| InDel-ED | 12 | 15,970,000 | 15,990,000 | 0.02 | | |
| InDel-ED | 12 | 16,020,000 | 16,050,000 | 0.03 | | |
| InDel-ED | 12 | 16,070,000 | 16,240,000 | 0.17 | | |
| InDel-ED | 12 | 16,260,000 | 16,360,000 | 0.1 | | |
| InDel-ED | 2 | 20,830,000 | 21,070,000 | 0.24 | | |
| InDel-ED | 2 | 21,090,000 | 21,090,000 | 0 | | |
| InDel-ED | 2 | 21,770,000 | 22,080,000 | 0.31 | | |
| InDel-ED | 3 | 15,070,000 | 15,420,000 | 0.35 | | |
| InDel-ED | 3 | 1,970,000 | 1,970,000 | 0 | | |
| InDel-ED | 3 | 1,990,000 | 2,250,000 | 0.26 | | |
| InDel-ED | 3 | 23,670,000 | 24,300,000 | 0.63 | | |
| InDel-ED | 4 | 32,560,000 | 32,570,000 | 0.01 | | |
| InDel-ED | 4 | 32,660,000 | 33,940,000 | 1.28 | | |

| | | | | |
|---|---|---|---|---|
| InDel-ED | 4 | 33,960,000 | 34,500,000 | 0.54 |
| InDel-ED | 4 | 34,580,000 | 34,680,000 | 0.1 |
| ΔInDel-index | 1 | 38,870,000 | 38,880,000 | 0.01 |
| ΔInDel-index | 1 | 38,910,000 | 40,340,000 | 1.43 |
| ΔInDel-index | 8 | 4,340,000 | 6,340,000 | 2.00 |
| ΔInDel-index | 8 | 6,380,000 | 6,480,000 | 0.1 |
| ΔInDel-index | 8 | 7,100,000 | 7,100,000 | 0 |
| ΔInDel-index | 8 | 7,270,000 | 7,400,000 | 0.13 |

**Supplementary Table S4 Candidate genes and gene annotation**

| Gene ID | Gene annotation |
|---|---|
| *LOC_Os08g08110* | diacylglycerol kinase, putative, expressed |
| *LOC_Os08g08120* | B-Box zinc finger gene; B-box (BBX) -containing protein |
| *LOC_Os08g08130* | transposon protein, putative, Pong sub-class, expressed |
| *LOC_Os08g08140* | receptor-like kinase, putative, expressed |
| *LOC_Os08g08150* | expressed protein |
| *LOC_Os08g08160* | expressed protein |
| *LOC_Os08g08170* | transposon protein, putative, CACTA, En/Spm sub-class |
| *LOC_Os08g08180* | retrotransposon protein, putative, Ty3-gypsy subclass |
| *LOC_Os08g08190* | expressed protein |
| *LOC_Os08g08200* | mRNA cap guanine-N7 methyltransferase 1, putative, expressed |
| *LOC_Os08g08205* | expressed protein |
| *LOC_Os08g08210* | H3K4-methytransferase; SET domain group protein; SET-domain-containing gene |
| *LOC_Os08g08220* | RING finger SNF2 ATPase |
| *LOC_Os08g08230* | kinesin-like protein, identical, putative, expressed |
| *LOC_Os08g08240* | transposon protein, putative, unclassified, expressed |
| *LOC_Os08g08250* | expressed protein |
| *LOC_Os08g08260* | expressed protein |
| *LOC_Os08g08270* | expressed protein |
| *LOC_Os08g08300* | transposon protein, putative, CACTA, En/Spm sub-class, expressed |
| *LOC_Os08g08310* | transposon protein, putative, CACTA, En/Spm sub-class, expressed |
| *LOC_Os08g08320* | expressed protein |
| *LOC_Os08g08330* | retrotransposon protein, putative, Ty3-gypsy subclass, expressed |
| *LOC_Os08g08340* | hypothetical protein |
| *LOC_Os08g08350* | retrotransposon protein, putative, Ty1-copia subclass, expressed |
| *LOC_Os08g08360* | expressed protein |
| *LOC_Os08g08370* | retrotransposon protein, putative, Ty1-copia subclass, expressed |
| *LOC_Os08g08380* | expressed protein |
| *LOC_Os08g08390* | retrotransposon protein, putative, unclassified, expressed |
| *LOC_Os08g08400* | retrotransposon protein, putative, unclassified, expressed |
| *LOC_Os08g08410* | retrotransposon protein, putative, Ty3-gypsy subclass, expressed |
| *LOC_Os08g08420* | expressed protein |
| *LOC_Os08g08430* | expressed protein |

| | |
|---|---|
| *LOC_Os08g08440* | expressed protein |
| *LOC_Os08g08450* | retrotransposon protein, putative, Ty3-gypsy subclass, expressed |
| *LOC_Os08g08460* | retrotransposon protein, putative, Ty3-gypsy subclass, expressed |
| *LOC_Os08g08500* | dihydroflavonol 4-reductase |
| *LOC_Os08g08510* | retrotransposon protein, putative, unclassified, expressed |
| *LOC_Os08g08520* | expressed protein |
| *LOC_Os08g08530* | retrotransposon protein, putative, unclassified |
| *LOC_Os08g08540* | retrotransposon protein, putative, unclassified, expressed |
| *LOC_Os08g08550* | retrotransposon protein, putative, Ty3-gypsy subclass, expressed |
| *LOC_Os08g08560* | retrotransposon protein, putative, unclassified, expressed |
| *LOC_Os08g08570* | expressed protein |
| *LOC_Os08g08580* | retrotransposon protein, putative, Ty3-gypsy subclass, expressed |
| *LOC_Os08g08586* | expressed protein |
| *LOC_Os08g08592* | expressed protein |
| *LOC_Os08g08600* | retrotransposon protein, putative, unclassified, expressed |
| *LOC_Os08g08610* | retrotransposon protein, putative, unclassified, expressed |
| *LOC_Os08g08620* | retrotransposon protein, putative, unclassified |
| *LOC_Os08g08630* | expressed protein |
| *LOC_Os08g08640* | retrotransposon protein, putative, Ty1-copia subclass, expressed |
| *LOC_Os08g08650* | expressed protein |
| *LOC_Os08g08655* | expressed protein |
| *LOC_Os08g08660* | expressed protein |
| *LOC_Os08g08670* | transposon protein, putative, CACTA, En/Spm sub-class, expressed |
| *LOC_Os08g08680* | transposon protein, putative, CACTA, En/Spm sub-class, expressed |
| *LOC_Os08g08690* | expressed protein |
| *LOC_Os08g08700* | ubiquitin family protein, putative, expressed |
| *LOC_Os08g08710* | expressed protein |
| *LOC_Os08g08720* | retrotransposon protein, putative, unclassified, expressed |
| *LOC_Os08g08730* | expressed protein |
| *LOC_Os08g08740* | expressed protein |
| *LOC_Os08g08750* | expressed protein |
| *LOC_Os08g08760* | ubiquitin family protein, putative, expressed |
| *LOC_Os08g08770* | retrotransposon protein, putative, Ty3-gypsy subclass, expressed |
| *LOC_Os08g08780* | retrotransposon protein, putative, Ty3-gypsy subclass, expressed |
| *LOC_Os08g08790* | ubiquitin family protein, putative, expressed |
| *LOC_Os08g08800* | expressed protein |
| *LOC_Os08g08810* | expressed protein |
| *LOC_Os08g08820* | GL2-type homeobox gene; Rice outermost cell-specific gene1 |
| *LOC_Os08g08830* | expressed protein |
| *LOC_Os08g08840* | glucose-6-phosphate/phosphate translocator 1 |
| *LOC_Os08g08850* | carotenoid synthesis regulator CarF, putative, expressed |
| *LOC_Os08g08860* | CRS2-associated factor 2, mitochondrial precursor, putative, expressed |
| *LOC_Os08g08870* | retrotransposon protein, putative, Ty1-copia subclass, expressed |
| *LOC_Os08g08880* | hypothetical protein |

| | |
|---|---|
| *LOC_Os08g08890* | expressed protein |
| *LOC_Os08g08900* | expressed protein |
| *LOC_Os08g08910* | hypothetical protein |
| *LOC_Os08g08920* | Cupin domain containing protein, Germin-Like Protein |
| *LOC_Os08g08930* | expressed protein |
| *LOC_Os08g08940* | transposon protein, putative, CACTA, En/Spm sub-class, expressed |
| *LOC_Os08g08950* | transposon protein, putative, CACTA, En/Spm sub-class, expressed |
| *LOC_Os08g08960* | germin-like protein |
| *LOC_Os08g08970* | Cupin domain containing protein; germin-like protein |
| *LOC_Os08g08980* | Germin-Like Protein |
| *LOC_Os08g08990* | Cupin domain containing protein, expressed |
| *LOC_Os08g09000* | Cupin domain containing protein, Germin-like protein |
| *LOC_Os08g09010* | Cupin domain containing protein, Germin-like protein |
| *LOC_Os08g09020* | Cupin domain containing protein, Germin-like protein |
| *LOC_Os08g09030* | hypothetical protein |
| *LOC_Os08g09040* | Cupin domain containing protein, Germin-like protein |
| *LOC_Os08g09050* | expressed protein |
| *LOC_Os08g09060* | Cupin domain containing protein, Germin-like protein |
| *LOC_Os08g09080* | Cupin domain containing protein, Germin-like protein |
| *LOC_Os08g09090* | hypothetical protein |
| *LOC_Os08g09100* | RNA recognition motif containing protein, putative, expressed |
| *LOC_Os08g09110* | Blast Resistance Gene 8 |
| *LOC_Os08g09120* | expressed protein |
| *LOC_Os08g09130* | conserved hypothetical protein |
| *LOC_Os08g09140* | retrotransposon protein, putative, Ty3-gypsy subclass, expressed |
| *LOC_Os08g09150* | retrotransposon protein, putative, unclassified, expressed |
| *LOC_Os08g09160* | retrotransposon protein, putative, unclassified, expressed |
| *LOC_Os08g09170* | expressed protein |
| *LOC_Os08g09180* | postsynaptic protein CRIPT, putative, expressed |
| *LOC_Os08g09190* | auxin efflux carrier component, putative, expressed |
| *LOC_Os08g09200* | aconitase |
| *LOC_Os08g09210* | glycinamide ribonucleotide synthetase |
| *LOC_Os08g09220* | OsFBX262 - F-box domain containing protein, expressed |
| *LOC_Os08g09230* | soluble starch synthase IIIa; white-core floury endosperm 5 |
| *LOC_Os08g09240* | autophagy associated gene |
| *LOC_Os08g09250* | glyoxalase I |
| *LOC_Os08g09260* | tRNA synthetase, putative, expressed |
| *LOC_Os08g09270* | pentatricopeptide repeat protein; chlorophyll deficient 4 |
| *LOC_Os08g09280* | hypothetical protein |
| *LOC_Os08g09290* | expressed protein |
| *LOC_Os08g09300* | expressed protein |
| *LOC_Os08g09310* | expressed protein |
| *LOC_Os08g09320* | YL1 nuclear protein C-terminal domain containing protein, expressed |
| *LOC_Os08g09340* | bromodomain containing protein, expressed |

| | |
|---|---|
| *LOC_Os08g09350* | gar2, putative, expressed |
| *LOC_Os08g09360* | expressed protein |
| *LOC_Os08g09370* | polyprenyl synthetase, putative, expressed |
| *LOC_Os08g09380* | OsFBX263 - F-box domain containing protein, expressed |
| *LOC_Os08g09390* | OsFBX264 - F-box domain containing protein, expressed |
| *LOC_Os08g09400* | expressed protein |
| *LOC_Os08g09410* | OsFBX265 - F-box domain containing protein, expressed |
| *LOC_Os08g09420* | OsFBX266 - F-box domain containing protein, expressed |
| *LOC_Os08g09430* | disease resistance protein RPM1, putative, expressed |
| *LOC_Os08g09440* | expressed protein |
| *LOC_Os08g09450* | OsFBL41 - F-box domain and LRR containing protein, expressed |
| *LOC_Os08g09460* | F-BOX PROTEIN 267 |
| *LOC_Os08g09470* | expressed protein |
| *LOC_Os08g09480* | OsFBX268 - F-box domain containing protein, expressed |
| *LOC_Os08g09490* | retrotransposon protein, putative, unclassified |
| *LOC_Os08g09500* | retrotransposon protein, putative, unclassified, expressed |
| *LOC_Os08g09510* | retrotransposon protein, putative, unclassified, expressed |
| *LOC_Os08g09520* | transposon protein, putative, unclassified, expressed |
| *LOC_Os08g09530* | retrotransposon protein, putative, Ty3-gypsy subclass |
| *LOC_Os08g09540* | hypothetical protein |
| *LOC_Os08g09570* | expressed protein |
| *LOC_Os08g09580* | hypothetical protein |
| *LOC_Os08g09590* | OsFBL42 - F-box domain and LRR containing protein, expressed |
| *LOC_Os08g09600* | F-box protein, putative, expressed |
| *LOC_Os08g09610* | expressed protein |
| *LOC_Os08g09630* | expressed protein |
| *LOC_Os08g09640* | OsFBL43 - F-box domain and LRR containing protein, expressed |
| *LOC_Os08g09650* | OsFBX269 - F-box domain containing protein, expressed |
| *LOC_Os08g09660* | G1 LIKE PROTEIN 12, G1-like 12; ALOG domain gene |
| *LOC_Os08g09670* | F-box/LRR-repeat protein 22, putative, expressed |
| *LOC_Os08g09680* | conserved hypothetical protein |
| *LOC_Os08g09690* | nuclear transcription factor Y subunit A |
| *LOC_Os08g09700* | OsFBX270 - F-box domain containing protein, expressed |
| *LOC_Os08g09710* | OsFBX271 - F-box domain containing protein, expressed |
| *LOC_Os08g09715* | F-box domain containing protein, expressed |
| *LOC_Os08g09720* | OsFBX272 - F-box domain containing protein, expressed |
| *LOC_Os08g09730* | OsFBX273 - F-box domain containing protein, expressed |
| *LOC_Os08g09740* | OsFBX274 - F-box domain containing protein, expressed |
| *LOC_Os08g09750* | F-box protein 3 |
| *LOC_Os08g09760* | OsFBX275 - F-box domain containing protein, expressed |
| *LOC_Os08g09770* | Endoplasmic Reticulum Chaperone |
| *LOC_Os08g09780* | expressed protein |
| *LOC_Os08g09790* | expressed protein |
| *LOC_Os08g09800* | WRKY105, expressed |

| | |
|---|---|
| *LOC_Os08g09810* | WRKY106, expressed |
| *LOC_Os08g09820* | peptidase aspartic family protein, putative, expressed |
| *LOC_Os08g09830* | BTB and MATH domain containing protein, putative, expressed |
| *LOC_Os08g09840* | WRKY117, expressed |
| *LOC_Os08g09850* | expressed protein |
| *LOC_Os08g09860* | hydroxyacid oxidase 1, putative, expressed |
| *LOC_Os08g09870* | expressed protein |
| *LOC_Os08g09880* | transposon protein, putative, unclassified, expressed |
| *LOC_Os08g09890* | expressed protein |
| *LOC_Os08g09900* | WRKY118, expressed |
| *LOC_Os08g09910* | OsFBX276 - F-box domain containing protein, expressed |
| *LOC_Os08g09920* | OsFBX277 - F-box domain containing protein, expressed |
| *LOC_Os08g09930* | expressed protein |
| *LOC_Os08g09940* | unconventional P-loop NTPase |
| *LOC_Os08g09950* | acyl-desaturase, chloroplast precursor, putative, expressed |
| *LOC_Os08g09960* | expressed protein |
| *LOC_Os08g12380* | retrotransposon protein, putative, Ty3-gypsy subclass, expressed |
| *LOC_Os08g12390* | retrotransposon protein, putative, Ty3-gypsy subclass, expressed |
| *LOC_Os08g12400* | retrotransposon protein, putative, Ty3-gypsy subclass, expressed |
| *LOC_Os08g12410* | pectinesterase, putative, expressed |

**Supplementary Table S5 The primers which were used in this study**

| Gene | Forward primer (5′-3′) | Reverse primer (5′-3′) |
|---|---|---|
| *UBQ* | GACGGACGCACCCTGGCTGACTAC | TGCTGCCAATTACCATATACCACGAC |
| *LOC_Os08g08970* | CTATGAATAAAGTCGGGTCT | CTCAGTAGCACGAGGATG |
| *LOC_Os08g09000* | GGATTTGCTTGCCTGGAC | GGTGAGTATGCGGTGGGT |
| *LOC_Os08g09900* | ACAACCACCCACGACAGT | CAGAAGCATTCCCATCAA |
| *LOC_Os08g09190* | CAAGGGTGTCGCTTATGT | TTTGAGTGCTCCGTTTCT |
